# Is the Satisfaction with Cancer Information Profile (SCIP) valid for tailoring information for patients with head and neck cancer?

**DOI:** 10.1186/1471-2407-8-164

**Published:** 2008-06-06

**Authors:** Matthew C Hankins, Carrie D Llewellyn

**Affiliations:** 1 Department of Primary Care & Public Health, Brighton & Sussex Medical School, Brighton, UK; 2 King's College London, Department of Psychology (at Guy's), Institute of Psychiatry, London, UK; 3 Brighton & Sussex University Hospitals NHS Trust, Brighton, UK

## Abstract

**Background:**

The Satisfaction with Cancer Information Profile (SCIP) has previously been shown to be a valid and reliable measure responsive to changes in patient satisfaction over time. It has been suggested that the SCIP might be used to guide the tailored provision of treatment information to patients with head and neck cancer but for this purpose the discrimination of the SCIP, not its responsiveness, should be assessed. This paper assesses whether the SCIP is valid as a discriminative measure suitable to guide tailored information.

**Methods:**

The SCIP comprises two parts (SCIP-A and SCIP-B). The discrimination of both parts was explored in a UK sample of 82 newly diagnosed patients with head and neck cancer. Principal components analysis (PCA) was first used to explore the factor structure of the SCIP-A and SCIP-B: discrimination analyses were then conducted at the level of full scale, subscale and item.

**Results:**

Principal components analysis revealed a coherent three-factor solution for the SCIP-A and a single factor for SCIP-B. Both parts of the SCIP proved to be discriminating at the full scale level (SCIP-A Delta = 0.92; SCIP-B Delta = 0.90). The SCIP-A also proved to be discriminating at the subscale level (Delta = 0.85 to 0.89). For the SCIP-A there was wide variation in the discrimination of individual items, confirming its potential to tailor information at the item level. For the SCIP-B, responses to most items indicated uniform satisfaction, suggesting that it would not be useful for tailoring information at the item level.

**Conclusion:**

The SCIP-A has been shown to be a valid discriminative measure and should prove suitable for tailoring treatment information at the level of item, subscale and total scale score. The SCIP-B, while a discriminating measure of total satisfaction, comprises too uniform a set of indicators of patient satisfaction to make it useful for tailoring information at the item level. Overall, the SCIP is valid as a measure of overall satisfaction with information about treatment and as a guide to tailoring such information.

## Background

In general, patients with cancer receive high-quality standard information [1] but often report a mismatch between their individual informational requirements and that actually provided [2-4]. It has been suggested that, while a common baseline level of information should be provided, patients would benefit from information provision tailored to their individual needs [5]. This suggestion has been supported by the National Cancer Alliance [6] which recognises that generic information might not fulfil the patients' requirements. Studies of patients with head and neck cancer (HNC) for example, revealed a need for more information about treatment options and the impact of treatment than the operation itself [7,8].

The assessment of quality of information can take many forms, from 'objective' evaluations of the accuracy of content, readability etc. [1] to more 'subjective' indices such as the patient's preference for [9,10] or satisfaction [11,12] with the information provided. While the former may have more relevance for issues surrounding patient knowledge and informed consent [13], the latter have been found to have an impact on clinical outcomes: for example, a recent study that found that lower levels of satisfaction with information about treatment were predictive of worse psychological outcomes in the longer term [3]. A recent review of the literature [4], however, found no assessment methods that took into account the amount, content and timing of the information about treatment for aspects such as recovery, side-effects and long-term consequences. Though some measures of informational need in cancer exist [14-16], they fail to capture the patient's perspective in terms of whether they have received too much or too little information and the level of information supplied. The recognition of the potential for different information needs for different types of cancer led to the development of the Satisfaction with Cancer Information Profile (SCIP) [17]. The SCIP has been validated as a reliable measure of satisfaction with information about treatment, responsive to change [3,17] and it has been suggested that this measure might guide a programme of tailored information provision. For the SCIP to be used for this purpose, however, requires that it be able to discriminate between different levels of individual satisfaction, and although validated in terms of being responsive to change, previous studies have not examined this aspect of the SCIP's validity. The SCIP might inform the selective provision of information to patients in two different ways. First, individual responses to the 14 items contained in the first subscale of the SCIP may be used to determine how information provision might be improved, as suggested by others [10]. For this purpose the discrimination of each item and the discrimination and reliability of the whole 14-item scale are of interest: if an individual item is insensitive to individual differences, it will be of limited use in tailoring interventions. Second, if there is an underlying structure to patient satisfaction with information (as has been reported for the Satisfaction with Information about Medicines Scale [18]), then information might be tailored on the basis of the level of satisfaction with each dimension. This might be required if resources do not allow for the tailoring of information to a precise (i.e. item-by-item) degree. For this purpose the discrimination and reliability of the dimension sub-scales would be of interest: if the sub-scales are unreliable or fail to discriminate between patients then they will be of doubtful value to inform focused interventions. This study therefore sought to validate the SCIP as a discriminative measure as a first step in establishing its usefulness as a guide to tailored information provision.

## Methods

### Sample

The database for this analysis was derived from a previously published study [17]. A subset of the database (SCIP item scores) was extracted for the full baseline sample of 82 patients newly diagnosed patients with HNC. These had been recruited into a prospective study from four hospitals in the southeast of England with a recruitment rate of 76%: there were no significant differences in age, gender or ethnicity between those recruited and those not recruited. The mean age was 60 (SD = 13) with a range of 23 to 89 years. The sample was predominantly white (92%) and male (66%), with most respondents married or cohabiting (61%). Approximately one half of the sample was diagnosed with early-stage disease (stages I and II: 48%) and one half with advanced-stage disease (stages III and IV: 46%). Stage at diagnosis was unobtainable for three patients. The most common sites were tongue (International Classification of Diseases [ICD]-10 C01 and C02) and laryngeal/glottis (ICD-10 C32). Initial treatment plans were varied: 27% surgery only; 26% radiotherapy only; 31% surgery and radiotherapy; 11% radiotherapy and chemotherapy and 5% surgery, radiotherapy, and chemotherapy (5%). SCIP data were obtained after diagnosis but before initial treatment.

### Analysis

The SCIP comprises two sections (here referred to as SCIP-A and SCIP-B for brevity). SCIP-A is a dichotomously scored (satisfied/not satisfied) 14-item scale while SCIP-B is a seven-item Likert-type scale (very dissatisfied to very satisfied) (see appendices A and B respectively for item listings). Because of the difference in scoring, different analyses were required:

#### SCIP-A reliability

The reliability of the SCIP-A was analysed using the KR-20 formula: this provides an estimate of the degree of measurement error in the scale score ranging from 1.0 (no error) to 0.0 (total error).

#### SCIP-A scale discrimination

Full scale discrimination was examined using Ferguson's Delta [19]. This provides an index of the degree of discrimination provided by the scale ranging from 0.0 to 1.0. It may be interpreted directly as the proportion of discriminations made in the sample, adjusted for the length of the scale. A Delta of 0.8, for example, means that 80% of all possible discriminations were made.

#### SCIP-A item analysis

The degree to which individual item responses predicted the scale score (the item-total correlation) was examined using the point-biserial correlation between each item and the scale score. The point-biserial correlation is simply the Pearson correlation coefficient between a continuous variable and a dichotomous variable and ranges from 0.0 (no association) to 1.0 (perfect prediction). Item discrimination was computed using the formula given by Allen & Yen [20]. This computes the difference in the number of endorsements of an item between the top and bottom thirds of the distribution, giving an index from 0.0 to 1.0. An index of 1.0 indicates perfect item discrimination, i.e. all of the most satisfied and none of least satisfied respondents endorsed the item. In addition, Ferguson's Delta was computed for each item, giving a second index of item discrimination.

#### SCIP-B reliability

The reliability of the SCIP-B was analysed using Cronbach's Alpha. This ranges from 0.0 to 1.0, with the same meaning and interpretation as the KR-20, above.

#### SCIP-B scale discrimination

Full scale discrimination was examined using Ferguson's Delta adapted for Likert-type scales [21]; this Delta also ranges from 0.0 to 1.0 and the meaning and interpretation are the same as Delta, above.

#### SCIP-B item analysis

item-total correlations for each item were computed using Pearson correlation coefficients. These range from 0.0 to 1.0 with the same meaning and interpretation as above. Item discrimination using the Allen & Yen formula was not possible as this formula is meaningful for dichotomous items only. However, item discriminations were computed using Delta, as above.

#### Sub-scale analysis

For both the SCIP-A and the SCIP-B the existence of meaningful subscales was explored using principal components analysis (PCA) with oblique rotation. This statistical method identifies components ('factors') of the scale scores. The exact number of factors was determined by examination of a 'scree' plot, which plots each factor identified against the variance explained by the factors. A levelling off of the curve suggests that further factors add little to the variance explained. Once identified, the nature of the components can be clarified by examining the association between each item and the factors ('item loadings') and the extent to which they explain the variation in scale scores (the 'explained variance'). Subscales may then be constructed from items having moderate to high loadings on a given factor. Sub-scales can be interpreted as measurements of different domains of satisfaction contributing to the overall satisfaction score. Since respondents may be more satisfied with one domain that another, they allow for discrimination at a level between full scale (total) satisfaction and the item-level satisfaction.

## Results

### Discrimination of SCIP-A

Table 1 shows the item analysis, reliability, and discrimination of SCIP-A. Since the items were dichotomous scores, the item means represent the proportion of respondents satisfied with each item. It can be seen that satisfaction ranged widely from item to item, with the greatest satisfaction expressed for information about ability to work and effects of treatment on appearance (item means = 0.81) and the least satisfaction expressed for information about what to do if experiencing side-effects (item mean = 0.56). Item-total correlations were also variable, ranging from 0.39 to 0.73, suggesting that items contributed to total satisfaction in varying degrees, as expected. Similarly, item discriminations ranged from 0.44 to 0.89; this indicates that some items were much more discriminating of high and low total satisfaction than others. For example, risk of complications and what to do if side effects were experienced had item discrimination scores of 0.89, indicating that satisfaction with these items was a good indicator of high overall satisfaction. Ability to work had an item discrimination score of 0.44, suggesting that it was very undiscriminating of total satisfaction. Item Delta coefficients assessed the ability of each item to discriminate between individuals, ranging from 0.60 to 0.98. The least discriminating items were ability to work and whether the treatment has any unwanted side effects (Delta = 0.60 and 0.62), while the most discriminating were sources of financial support (Delta = 0.98), what to do if experiencing side-effects and impact on quality of life (QoL) (both Delta = 0.95). Scale reliability and scale discrimination were good (Alpha = 0.89; Delta = 0.92).

**Table 1:** Item analysis, reliability and discrimination for SCIP-A

**Item**	**Item mean**	**Item-total correlation**	**Item discrimination**	**Item Delta**
1. Unwanted side-effects	0.78	0.63	0.61	0.62
2. Risks of side-effects	0.74	0.73	0.72	0.76
3. Risks of complications	0.59	0.66	0.89	0.92
4. Experience of side-effects	0.56	0.64	0.89	0.95
5. Interferes with medication	0.72	0.39	0.56	0.84
6. Feel after treatment	0.78	0.68	0.61	0.68
7. Ability to work	0.81	0.52	0.44	0.60
8. Financial support	0.61	0.41	0.61	0.98
9. Further treatment	0.78	0.59	0.61	0.74
10. Effect on appearance	0.81	0.59	0.50	0.69
11. Long term impact	0.76	0.50	0.50	0.80
12. Recovery time	0.67	0.53	0.61	0.89
13. Impact on QoL	0.61	0.63	0.78	0.95
14. Patient support	0.69	0.63	0.72	0.93

Scale KR-20	0.89			
Scale Delta	0.92			

### Factor structure of SCIP-A

Principal components analysis of the SCIP-A with oblique rotation initially revealed 4 factors, with Eigen values greater than 1, accounting for 61.7% of total variance. Inspection of the fourth factor revealed that it comprised two items, both of which loaded on one of the other three factors: the scree plot also suggested a three factor solution explaining 59.6% of total variance (Table 2). The first factor accounted for 40.9% of total variance and was interpreted as satisfaction with information about *unwanted effects *(items 1,2,3,4,5; Alpha = 0.83; Delta = 0.87). The second factor accounted for 10.6% of total variance and was interpreted as satisfaction with information about *long-term effects *(items 6,9,10,11,12,13; Alpha = 0.85; Delta = 0.85). The third factor accounted for 8.1% of total variance and was interpreted as satisfaction with information about *social/financial support *(items 7,8,14; Alpha = 0.66; Delta = 0.89). The three subscales were significantly correlated (unwanted effects and long-term effects r = 0.56, p < 0.001; unwanted effects and financial/social support r = 0.56, p < 0.001; long term effects and financial/social support r = 0.52, p < 0.001).

**Table 2:** SCIP-A factor loadings following PCA

**Item**	**Item content**	**Rotated on-factor loadings**
**Subscale 1: 'Unwanted effects'**		
**1**	Whether the treatment has any unwanted side-effects	0.77
**2**	What the risks of your experiencing side-effects are	0.75
**3**	What the risks of your experiencing complications are	0.75
**4**	What you should do if you experience unwanted side-effects	0.88
**5**	Whether your treatment interferes with other medicines you may be taking	0.21
**Subscale 2: 'Long-term effects'**		
**6**	How you may expect to feel immediately after treatment	0.55
**9**	Whether you may need further treatment in the future	0.63
**10**	The effect of treatment on your appearance	0.38
**11**	The long-term impact of treatment on functioning (daily activities)	0.60
**12**	How long you expect recovery to take	0.89
**13**	How your treatment may impact on your quality of life over the next year	0.71
**Subscale 3: 'Social/financial support'**		
**7**	The effects of treatment on your ability to work	0.59
**8**	Who to ask/where to go for possible financial support	0.85
**14**	Patient support groups for you and your partner	0.61

### Discrimination of SCIP-B

The reliability, discrimination and item analysis of the SCIP-B are reported in Table 3. In general patients were satisfied with the type of information received and the timing, with mean item scores ranging from 3.7 to 4.2 (i.e. 'satisfied' on the five point integer scale). Item-correlations were also high ranging from r = 0.52 to r = 0.74. There was wide variability in individual items, however, with standard deviations ranging from 0.5 to 1.0, suggesting that some items might be more discriminating than others. This was confirmed by the item Delta coefficients ranging from low (usefulness and detail of information; Delta = 0.71) to highly discriminating (written information; Delta = 0.93). Scale reliability and discrimination were satisfactory (Alpha = 0.87; Delta = 0.90).

**Table 3:** Item analysis, reliability and discrimination for SCIP-B

**Item**	**Item mean**	**Item SD**	**Item-total correlation**	**Item Delta**
Usefulness of information to you	4.1	0.6	0.74	0.71
Usefulness to others	4.0	0.7	0.69	0.78
Written information	3.7	1.0	0.52	0.93
Verbal information	4.2	0.7	0.76	0.72
Timing of information	4.0	0.8	0.63	0.73
Detail of information	4.1	0.7	0.69	0.71
Understanding of information	4.2	0.5	0.67	0.54

Scale Alpha	0.87			
Scale Delta	0.90			

### Factor structure of SCIP-B

Principal components analysis with oblique rotation suggested a single factor accounting for 61.0% of the total variance (Table 4). Examination of the scree plot also suggested a one-factor solution as the best fit. The SCIP-B was therefore determined to be one-dimensional and no division into subscales was necessary.

**Table 4:** Items included in SCIP-B with results from PCA

**SCIP no.**	**Item content**	**Rotated on-factor loadings**
**15**	The usefulness of the information to you	0.87
**16**	The usefulness of the information to your partner/family	0.85
**17**	The amount of written information supplied	0.82
**18**	The amount of verbal information supplied	0.79
**19**	The timing at which you received information	0.78
**20**	The detail of the information given to you	0.75
**21**	How understandable the information was to you	0.56

## Discussion

For a measure to be useful in guiding tailored information it must be able to discriminate between the differing needs of individuals on a number of levels. Both sections of the SCIP showed good discrimination at the level of total satisfaction (SCIP-A Delta = 0.90; SCIP-B Delta = 0.93), demonstrating that the measures should prove useful in determining which patients are in need of further information in general. Additionally the SCIP-A further subdivided into three coherent, correlated but distinct areas of satisfaction (unwanted effects, long-term effects and financial/social support). While the first two of these subscales had acceptable reliability and all had adequate discrimination, the financial support subscale was low in reliability. Since this subscale comprised only three items, this is perhaps not surprising: further development in measuring this aspect of patient satisfaction is required. However, it seems likely that the three subscales will also prove useful as discriminating measures of patient satisfaction within the broad domains so identified.

At the most patient-specific level of discrimination (discrimination by item) some items proved more discriminating than others, particularly those of the SCIP-A. This justifies the consideration of item-level responses, since total satisfaction score was not consistently related to item scores, and suggests that these items will be the most effective in identifying areas for additional or tailored provision of information.

As expected, those items uniformly high in satisfaction across the sample were less useful in specifying how information provision might be improved: this was especially the case for the SCIP-B as the result of a 'ceiling effect' for all of the items except the provision of written information. Questionnaires measuring general or overall satisfaction often suffer from this same lack of variability and it may be that the response scale of SCIP-B should be further adapted to increase the spread of scores [22].

We envisage that the SCIP will be useful in clinical settings, research and audit. As a discriminating measure, it should prove possible to tailor information provision at the individual or group level, with the SCIP administered immediately after the initial consultation, or indeed at any stage in the course of treatment. For research, the SCIP has now been validated for discriminative and evaluative purposes and has been shown to be sensitive to individual differences and change over time. For audit, the patient's overall level of satisfaction with information provision may be assessed, and once again, changes in satisfaction during the audit cycle noted.

The result of this study may, of course, reflect the particular concerns and response characteristics specific to patients with head and neck cancer. It is possible that some or all of these concerns extend to patients with other forms of cancer, but we suggest that the SCIP be piloted and further adapted (if required) before use in studies of different populations.

## Conclusion

The SCIP proved to be a reliable and discriminating measure of satisfaction suitable for guiding the tailoring of information for patients with head and neck cancer. The SCIP-A may be used to tailor the provision of information based on total satisfaction score, subscale scores and item scores. The SCIP-B is better suited to tailoring information at the level of total satisfaction since it appears to be uni-dimensional and comprises items with low individual discrimination.

## Competing interests

The authors declare that they have no competing interests.

## Authors' contributions

MCH came up with the idea for the manuscript, performed data analyses and interpretation and wrote the manuscript. CDL designed and conducted the original study and helped to draft the manuscript. Both authors approved the final manuscript.

## Appendix: The SCIP questionnaire items and scoring

### SCIP A

Do you feel as if you have received enough information about:

1. Whether the treatment has any unwanted side effects

2. What the risks of your experiencing side effects are

3. What the risks of your experiencing complications are

4. What you should do if you experience unwanted side effects 0 1 0 1

5. Whether your treatment interferes with other medicines you may be taking

6. How you may expect to feel immediately after treatment

7. The effects of treatment on your ability to work

8. Who to ask/where to go for possible financial support

9. Whether you may need further treatment in the future

10. The effect of treatment on your appearance

11. The long term impact of treatment on functioning (daily activities)

12. How long you expect recovery to take

13. How your treatment may impact on your quality of life over the next year

14. Patient support groups for you and your partner

Response choices: Too much/About right/Too little/None wanted

Scoring: 0/1/0/1

Possible subscale score: 0–14

### SCIP-B

Overall, how would you rate the following?

1. The usefulness of the information to you

2. The usefulness of the information to your partner/family

3. The amount of written information supplied

4. The amount of verbal information supplied

5. The timing at which you received information

6. The detail of the information given to you

7. How understandable the information was to you

Response choices: Very satisfied/Satisfied/Neither/Dissatisfied/Very dissatisfied

Scoring: 5/4/3/2/1

Possible subscale score: 7–35

## Pre-publication history

The pre-publication history for this paper can be accessed here:


